# Environmental degradation and the increasing burden of allergic disease: The need to determine the impact of nitrogen pollution

**DOI:** 10.3389/falgy.2023.1063982

**Published:** 2023-02-02

**Authors:** Tobias Ceulemans, Paulien Verscheure, Caroline Shadouh, Kasper Van Acker, Brecht Devleesschauwer, Catherine Linard, Nicolas Dendoncker, Niko Speybroeck, Nicolas Bruffaerts, Olivier Honnay, Rik Schrijvers, Raf Aerts

**Affiliations:** ^1^ Department Biology, UAntwerpen, Antwerpen, Belgium; ^2^ Division Ecology, Evolution, and Biodiversity Conservation, KU Leuven, Leuven, Belgium; ^3^ KU Leuven Department of Microbiology, Immunology and Transplantation, Allergy and Clinical Immunology Research Group, KU Leuven, Leuven, Belgium; ^4^ Institut de Recherche Santé et Societé, UC Louvain, Louvain-la-Neuve, Belgium; ^5^ Department of Epidemiology and Public Health, Sciensano, Brussels, Belgium; ^6^ Department of Translational Physiology, Infectiology, and Public Health, Ghent University, Merelbeke, Belgium; ^7^ Department of Geography, University of Namur, Namur, Belgium; ^8^ Mycology and Aerobiology, Sciensano, Brussels, Belgium; ^9^ Risk and Health Impact Assessment, Sciensano, Brussels, Belgium

**Keywords:** allergic disease, biodiversity loss, environmental degradation, environmental pollution (including CO_2_ and SO_2_ emissions), epidemiology, nitrogen deposition, pollen allergy, respiratory health

## The increasing burden of pollen allergy

Allergic rhinitis, conjunctivitis, and asthma are common manifestations of allergies (1). These allergic disorders are associated with a relative high health burden for modern societies because of their high prevalence, often life-long morbidity, and impact on mental health and well-being. Therefore, they are a major public health problem (2). The past decades, the allergy prevalence and sensitization rates have globally increased (1, 3–7). This prevalence is expected to even further increase in the following decades.

On the one hand, the increase is expected through a better general population understanding of allergy that will increase the number of patients seeking help (8). Also, the implementation of better diagnostic methods may increase the number of patients getting diagnosed. Even so, an increasing prevalence over the years has already been observed with traditional skin-prick testing, a longstanding standard of care for respiratory allergy diagnosis (7). On the other hand, a rise in allergic diseases is also expected as a result of various interactions between changes in the environment and modern lifestyle (1, 9–11).

One such interaction is the air pollution, that accompanies the global increase in urbanization, which exacerbates airway diseases including allergic rhinitis as a consequence of pollen exposure (12). Several mechanisms have been described by which air pollution can affect pollen allergens and their ability to induce allergenic symptoms in patients. Firstly, rising environmental ozone (O_3_) and ambient CO_2_ concentrations have been documented to alter allergenic properties of pollen grains in grasses and in tree species (4, 13–15). Secondly, air pollutants can also cause respiratory inflammation and epithelial barrier damage, therefore priming the airways and facilitate allergen access (16). Furthermore, brief exposure to nitrogen dioxide (NO_2_) has resulted in an enhanced eosinophilic activity in response to allergens by pollen-allergenic asthmatics (17). Also, nitrogen dioxide exposure in a mouse model was able to induce an allergenic response to aerosolized OVA, an innocuous protein that by itself does not trigger an immune response (18). Pollen exposed to ozone and NO_2_ have also shown an increased activation of oxidative defense mechanisms which was associated with increased IgE recognition (19). In addition, air pollutants can affect allergen protein structure by inducing post-translational modifications e.g., protein nitration, leading to increased allergen specific IgE levels (20, 21). Finally, air pollutant exposure has been associated with sensitization to pollen allergens and with pollen-allergic symptoms (22–25).

These effects might be species dependent and while decreasing the pollen load for some species, it might also increase for others. Allergic sensitization in patients also results in different seasonal profiles and varies among different regions. The overall effect can result in pollen allergy patients that are exposed to more of certain pollen types and potentially increasingly potent pollen and this often over longer time periods. This escalates the burden of allergic diseases and the costs for society. Additionally, in the context of the recent global pandemic, pollen allergy patients may be more susceptible to infectious diseases including COVID-19, as pollen exposure weakens the innate defense against respiratory viruses (26) and unmanaged hay fever may increase the risk of viral dissemination, for instance through excessive sneezing (27).

## Environmental nitrogen pollution causing increasing prevalence of allergy?

The risk for respiratory allergic diseases may also be exacerbated by global environmental change. Indeed, environmental change can alter plant species distribution ranges, pollen amounts and pollen traits, the onset and length of pollen seasons, and the atmospheric pollen distribution patterns (7, 10, 28–30). For instance, rising global temperatures have caused long-term changes in the timing and increases in the duration and the intensity of a series of allergenic pollen seasons in Europe, for instance for birch, alder and hazel trees (31–34). Thus, depending on the plant species and their susceptibility to climatic forces, the duration and intensity of human pollen exposure has changed.

A potentially important yet underexplored global change driver that may drive increasing aeroallergens and allergy prevalence is *environmental nitrogen pollution*. Emissions from the combustion of fossil fuels and intensive agriculture have caused a twofold increase of biologically reactive nitrogen worldwide (35, 36). The anthropogenic perturbation of the global nitrogen cycle is therefore identified as one of the largest threats to the global biosphere integrity and human health (37–40). In high nitrogen emission regions, total inorganic nitrogen deposition rates are well above 50 kg N ha^−1^ yr^−1^ (41). In the Atlantic biogeographic region of Europe, current atmospheric nitrogen deposition levels range between 2.4 and 43.5 kg N ha^−1^ yr^−1^ depending on land use and land cover (42), with the highest levels associated with intensive agriculture, urbanization, and industry (43). The health risks associated with atmospheric nitrogen pollution, such as the risks of allergic inflammatory diseases and asthma associated with atmospheric NOx, are well recognized (44–48). However, the potential impacts of nitrogen pollution on human health *via* increased soil nitrogen availability and subsequent plant uptake of nitrogen, have not yet been quantified and the mechanisms are poorly understood (39, 49, 50). Yet, here we argue that this may represent important unexplored pathways for increased pollen allergy prevalence.

The ecological effects of environmental nitrogen enrichment include biodiversity loss, changes in plant community composition, ecosystem simplification, and loss of ecosystem service provisioning capacity (51–54). Specifically, nitrogen pollution leads to more productive ecosystems, which tend to become species-poor and dominated by a few, highly competitive plant species (52, 55–57). For example, in acidic grasslands surveyed across a nitrogen deposition gradient in the Atlantic biogeographic region of Europe, plant species richness decreased by 21.7% for each increment of 10 kg total inorganic nitrogen deposition ha^−1^ yr^−1^ [OR = 0.78 (42)]. Importantly in the context of pollen allergy, nitrogen pollution in heathlands and grasslands worldwide is associated with an increase in productivity of grasses (for instance *Molinia caerulea*) that become dominant at the expense of forbs and dwarf shrubs (52, 58–59). As grass pollen are among the world's most harmful aeroallergens (60), it is easy to conceive that nitrogen pollution may thus change pollen allergenic landscapes to the detriment of the societal burden of respiratory disease. In forests also, nitrogen pollution can cause an increase in tree productivity (61), that subsequently may also lead to increases in airborne tree pollen and the burden of allergy.

It has been hypothesized before that changes in plant community composition and primary productivity may affect airborne pollen distributions and abundances with subsequent potential knock-on effects for pollen allergy patients (60, 62). However, to our knowledge, changes in plant community composition and productivity driven by environmental nitrogen pollution remain to be linked with changes in the severity of pollen allergy. Yet, in an era of ever increasing nitrogen pollution (39, 49, 50) and of allergic disorders (1, 3–6), we feel that it is urgently needed to quantify this pathway.

In addition to changes in plant communities driven by nitrogen pollution, experiments have shown that the biochemical composition of plant pollen is significantly altered following nitrogen fertilization (63, 64). These biochemical changes may very well affect the allergen potency of pollen with important human health consequences, even if plant pollen productivity and community composition of allergenic plant species would be unaffected by nitrogen pollution. Unfortunately, data on biochemical changes in pollen following nitrogen enrichment is very scarce and the potential effect on allergy response remains a broad knowledge gap. We hypothesize that nitrogen-induced biochemical changes in pollen allergenicity could be associated with more severe allergy symptoms, indicated by increased IgE-specific reactivity (*in vitro*) or increased allergy symptom severity scores (*in vivo*).

**Figure 1:**
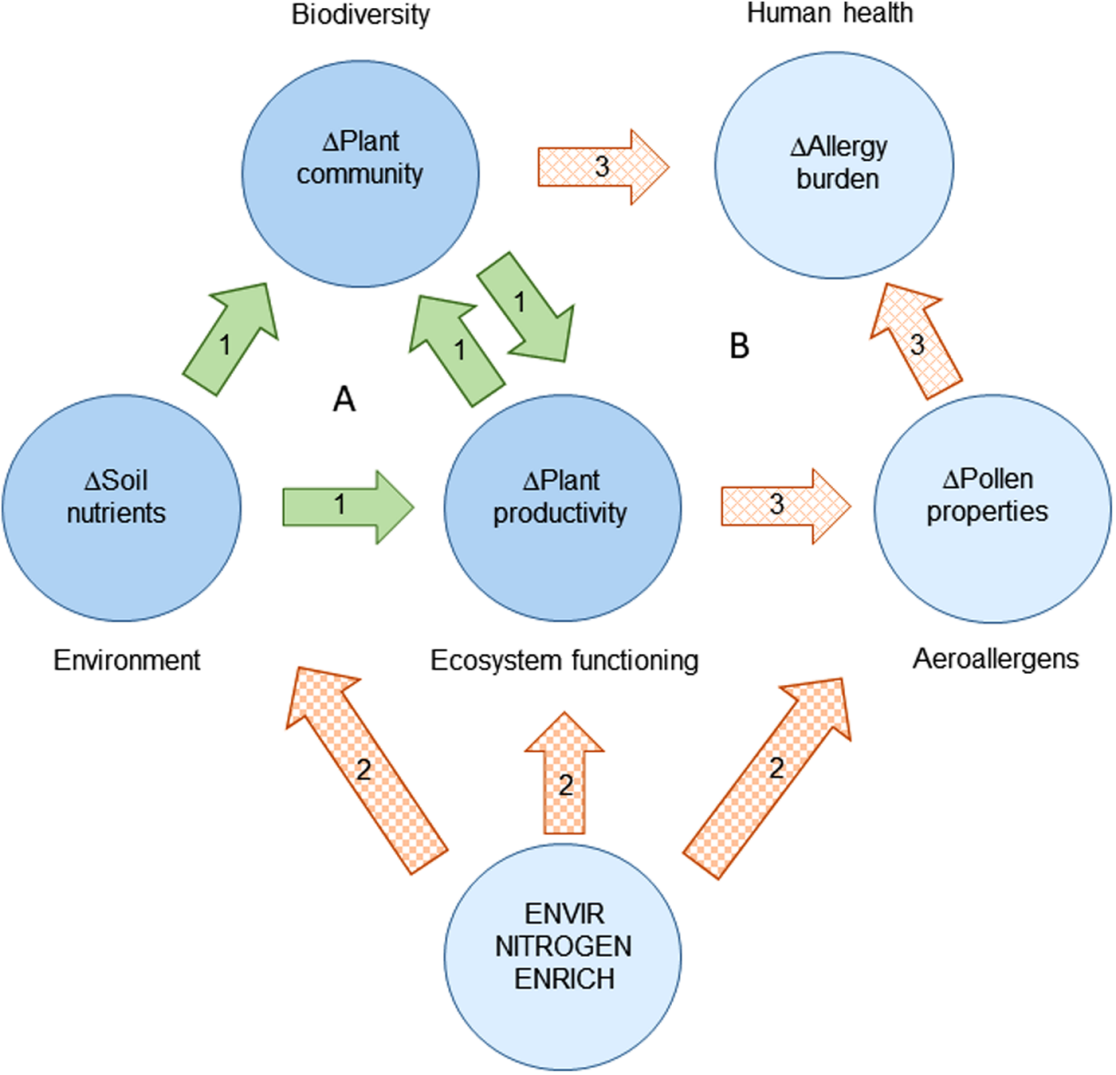
Environmental nitrogen pollution and the burden of allergic disease in the light of environment, biodiversity, and ecosystem functioning. (**A**) Under the present “biodiversity-ecosystem functioning” paradigm, biodiversity and environmental variation drive ecosystem functioning, such as plant productivity (arrow set 1). (**B**) Environmental nitrogen pollution may drive changes in the burden of pollen allergies through a combination of direct changes (arrow set 2) and indirect changes (arrow set 3) in the abiotic environment, plant species composition, plant productivity, airborne pollen species composition and abundance, and, importantly, allergen potency.

Nitrogen-induced changes in plant species composition, in combination with altered allergenic properties of plants, could therefore result in landscapes with elevated allergy risks relative to landscapes free of nitrogen pollution. In addition, the combination of higher allergy risks at the level of the landscape and increased allergy symptom severity escalates the burden of allergic disease (Figure 1).

## Conclusion

Environmental nitrogen pollution may have important direct and indirect impacts on plant species composition and productivity on the one hand, and on allergenicity of pollen aeroallergens on the other. Consequently, nitrogen pollution may change the prevalence, incidence, and severity of allergic disease by modifying the places where people live into landscapes with higher pollen exposure, resulting in elevated allergy risks, and increased allergy symptom severity.

Nitrogen pollution worldwide urges us to determine the effects on allergy prevalence, allergenicity and symptom severity to understand, prevent, and control these unexplored pathways of nitrogen-driven allergy risks to public health.

We argue that these insights are needed to better inform environmental policy with respect to reduction of environmental nitrogen pollution, which at present only aims to reduce the direct impact of air pollution on respiratory health and to support biodiversity conservation. It does not take into account the potential effects on public health we have outlined here. Yet, improved policy measures may not only help to maintain the favourable conservation status of biodiversity and vulnerable habitats, but also help to sustain the habitability of areas prone to nitrogen pollution, such as urbanized and industrialized regions and regions with intensive agriculture—indeed the places where most people live.
